# Supplementary material for: Comparison of Illumina versus Nanopore 16S rRNA Gene Sequencing of the Human Nasal Microbiota
Source: Genes (Basel). 2020 Sep 21;11(9):1105. doi: 10.3390/genes11091105 (PMC7565314; doi:10.3390/genes11091105)
Supplement: Supplementary file 1 [file genes-11-01105-s001.pdf]

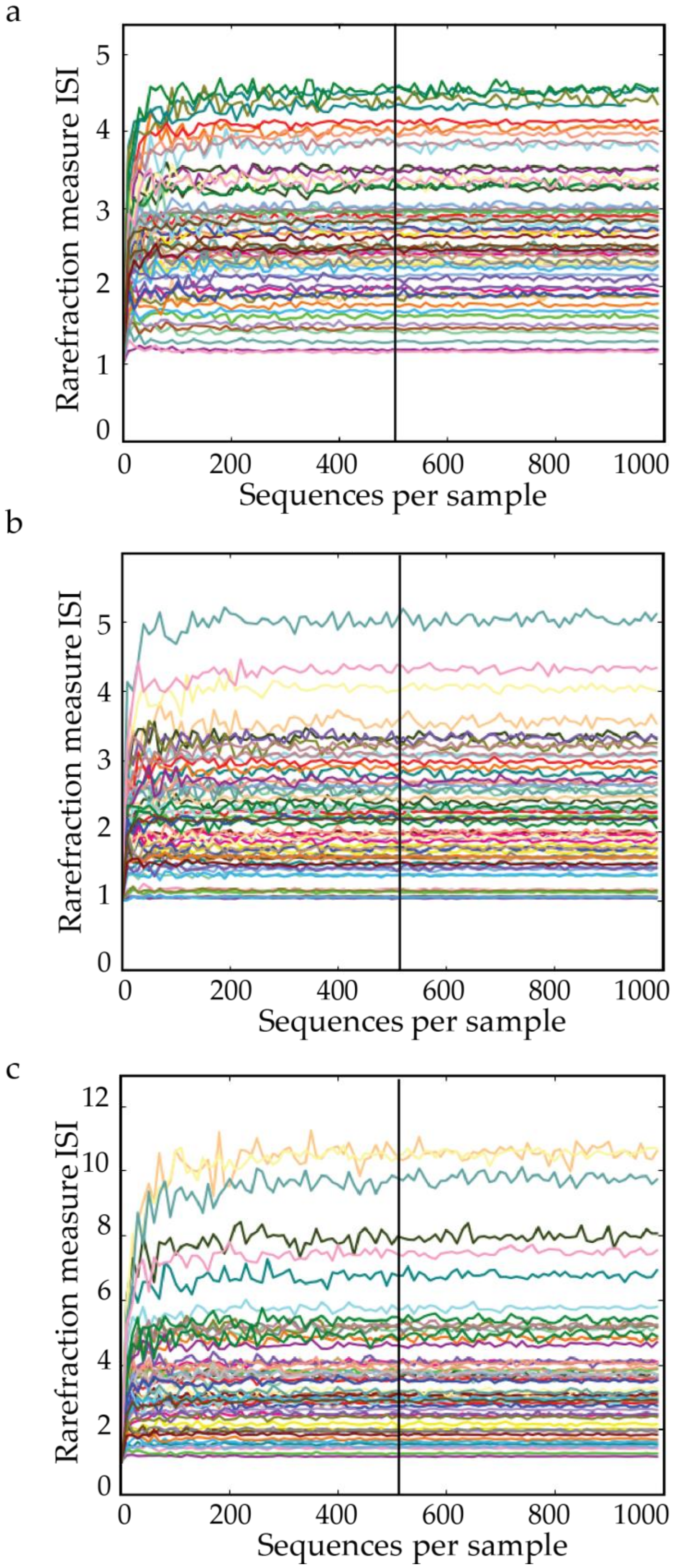
a
Rarefraction measure ISI
Sequences per sample
0
1
2
3
4
5
0
200
400
600
800
1000
b
Rarefraction measure ISI
Sequences per sample
0
1
2
3
4
5
0
200
400
600
800
1000
c
Rarefraction measure ISI
Sequences per sample
0
2
4
6
8
10
12
0
200
400
600
800
1000

Figure S1.

Rarefaction curves of sequenced nasal swab samples.

Plots were generated with QIIME 1.9.1 (multiple_rarefactions.py, alpha_diversity.py, collate_alpha.py, make_rarefaction_plots.py) using the Shannon diversity metric. See https://bioinf-galaxian.erasmusmc.nl/public/astrid/qiime/makeplots.sh for full script. (**a**) determined by Illumina sequencing at genus level (**b**) determined by nanopore sequencing at genus and (**c**) species level.

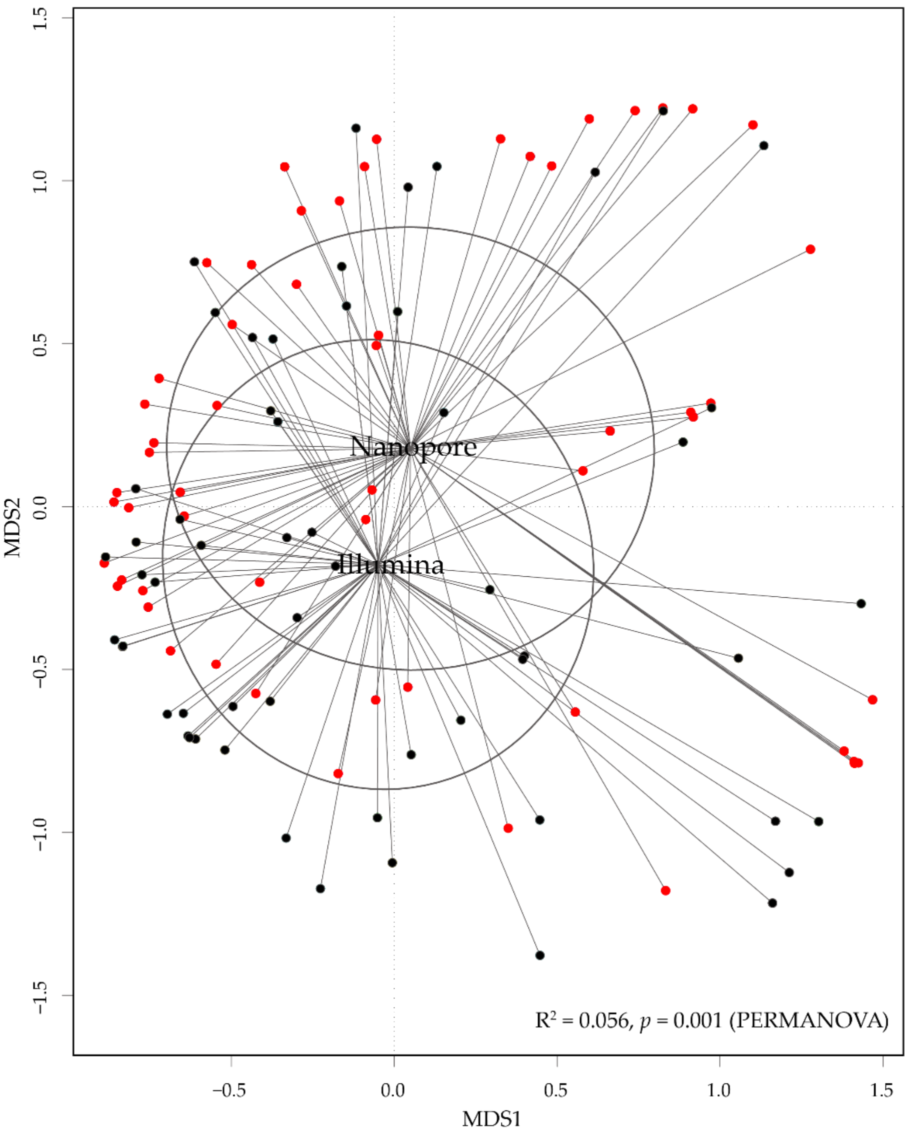

Figure S2.

Principle coordinate analysis on Illumina and nanopore nasal microbiota profiles. To determine the variability in microbiota profiles between the Illumina and the nanopore platform, principal coordinate analysis and PERMANOVA statistics (vegan, R) was performed on the seven most prevalent genera, the unclassified reads and the remaining

genera combined as others. The platforms only contributed 5.6%, $p = 0.001$ to the total variation in taxonomy data, demonstrating that the platforms performed similarly.

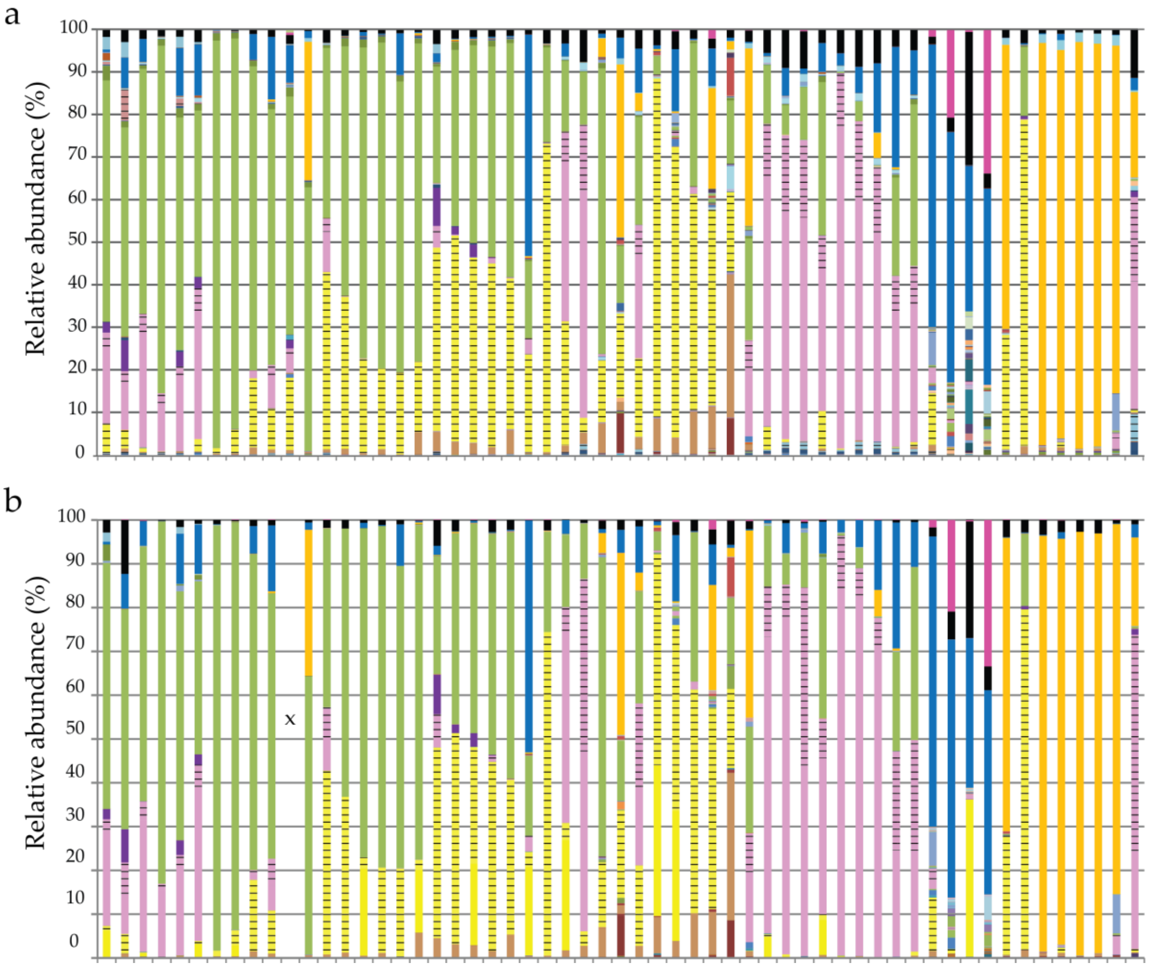

Figure S3.
Re-basecalling of nanopore sequence reads derived from nasal swabs.
DNA was isolated from 57 nose swab samples and 16S rRNA gene sequencing was performed using the Oxford Nanopore sequencing platform. The sequence reads were basecalled and analysed twice, using the Albacore basecaller and the EPI2ME versions 2.47.537208 or 2.48.690655 16S pipeline (**a**), or the Guppy basecaller and the EPI2ME version 2020.2.10 16S pipeline (**b**). Each bar in the graph represents a nasal microbiota profile from a single individual, with a similar sample order in (**a**) and (**b**). The dashed lines in (**a**) and (**b**) represent genera that, by default, were reported as unclassified at genus level in the EPI2ME report but were identified when, next to reads with a top three blast hit with similar genera (num_genus_taxid is 1) (**a**), or lca is 0 (**b**), reads with a top three blast hit with two genera (num_genus_taxid is 2) (**a**), or lca of 1 and a top

BLAST identification of *Dolosigranulum* sp. or *Heamophilus* spp. (**b**) were included. x is insufficient read numbers remained for this sample (sample 16) after basecalling.
